# ﻿The complete mitochondrial genome of the terrestrial snail *Monachacartusiana* (O.F. Müller, 1774) (Gastropoda, Eupulmonata, Hygromiidae)

**DOI:** 10.3897/zookeys.1130.91325

**Published:** 2022-11-17

**Authors:** Ewa Kosicka, Joanna R. Pieńkowska, Andrzej Lesicki

**Affiliations:** 1 Department of Cell Biology, Institute of Experimental Biology, Faculty of Biology, Adam Mickiewicz University in Poznan, Uniwersytetu Poznańskiego 6, 61-614, Poznań, Poland Adam Mickiewicz University Poznań Poland; 2 Department of Bioenergetics, Institute of Molecular Biology and Biotechnology, Faculty of Biology, Adam Mickiewicz University in Poznan, Uniwersytetu Poznańskiego 6, 61-614, Poznań, Poland Adam Mickiewicz University Poznań Poland

**Keywords:** Carthusian snail, Helicoidea, mitogenome, phylogeny, Stylommatophora

## Abstract

The mitochondrial genome of *Monachacartusiana* is the first complete mitochondrial sequence described for the pulmonate snail genus *Monacha* and for the family Hygromiidae. The identified mitogenome has a length of 13,894 bp and encodes 13 proteins, 22 tRNAs, and two rRNAs. A phylogenetic analysis of available mitogenomes from representatives of helicoid families shows a sister group relationship of Hygromiidae and Geomitridae, which have been recently recognised as separate families.

## ﻿Introduction

Mollusca is the second largest animal phylum after Arthropoda in terms of the number of named species, with the class Gastropoda as the most speciose group with approximately 95,000 species (Ponder et al. 2020). Stylommatophoran pulmonates constitute the most species-rich gastropod order with an estimated number of about 30,000 species (Mordan and Wade 2008). Although the monophyly of Stylommatophora within panpulmonate heterobranchs is relatively well established (Jörger et al. 2010; Ponder et al. 2020), the phylogenetic relationships between stylommatophoran families are still debated (White et al. 2011; Gaitán-Espitia et al. 2013; Razkin et al. 2015; Doğan et al. 2020; Ponder et al. 2020).

Mitogenome sequences are of great importance in molecular phylogenetic studies (Moritz et al. 1987), especially to infer evolutionary relationships at species level (Avise et al. 1987); this is also the case within Mollusca (Boore 1999). The analysis of mito­genomes may thus provide additional evidence related to stylommatophoran phylogeny (White et al. 2011; Parmakelis et al. 2013; Minton et al. 2016a). The number of available stylommatophoran mitogenomes has increased in the last two decades, from three at the end of the 20^th^ century (Hatzoglou et al. 1995; Terrett et al. 1996; Yamazaki et al. 1997) to 35 in recent years (Yang et al. 2019; Doğan et al. 2020). However, considering the number of recognised extant families within the Stylommatophora (117 according to Bouchet et al. 2017), the number of stylommatophoran mitogenomes still is very small and new mitogenomes, especially from families for which no, or very few, mito­genomes are available, are worth publishing. Hitherto, two mitogenomes were available for the Hygromiidae, viz. *Cernuellavirgata* (Da Costa, 1778) and *Helicellaitala* (Linnaeus, 1758), published by Lin et al. (2016) and Romero et al. (2016), respectively. However, these two species have recently been transferred from the Hygromiidae to the Geomitridae (Razkin et al. 2015; Neiber et al. 2017; Bouchet et al. 2017), so that the Hygromiidae, very rich in species, is left without any available mitogenome.

The hygromiid genus *Monacha* Fitzinger, 1833 is widespread in the western Palae­arctic from western Europe to North Africa, Iran, and Arabia. It includes a large number of nominal species and shows its highest diversity in south-eastern Europe and Turkey (Hausdorf 2000a, 2000b; Welter-Schultes 2012). Although most of the *Monacha* species occur in rather narrow areas (Welter-Schultes 2012; Neiber and Hausdorf 2017), *Monachacartusiana* (O.F. Müller, 1774), the type species of the genus, is widely distributed and can be found in almost the whole of Europe excluding its north-eastern fringes (Scandinavia, Russia, Baltic States, Belarus, northern Ukraine) (Welter-Schultes 2012; Pieńkowska et al. 2018). The mitogenome of this species will facilitate the future identification of species within the genus and the understanding of their phylogenetic relationships, as is the case with other families of terrestrial pulmonate snails (González et al. 2016; Groenenberg et al. 2017; Korábek et al. 2019; Doğan et al. 2020). Hence, in this paper, we present the complete mitogenome of *M.cartusiana* and analyse its phylogenetic position within the superfamily Helicoidea.

## ﻿Material and methods

The specimen of *Monachacartusiana* used for this research was collected in Ostrowiec Świętokrzyski (Poland) by Mariusz Gwardjan on 03.07.2015. It was identified by the sequence of the cytochrome c oxidase subunit I gene fragment (*coI*) of *M.cartusiana* in GenBank (KX258398) deposited by Pieńkowska et al. (2016). Total genomic DNA was extracted following Pieńkowska et al. (2015). The sequencing of the *M.cartusiana* mitogenome (for gene acronyms see Table 3) was started using four pairs of primers complementary to the conservative regions of *coI* (Folmer et al. 1994), *16S rRNA* (Palumbi et al. 1991), *coII* (Hugall et al. 2002) and *cytb* (Merritt et al. 1998), the missing fragments between them were identified by primer walking (Lin et al. 2016). The primers used for the amplification of mtDNA are listed in Table 1.

**Table 1.** List of primers used for the amplification of *Monachacartusiana* mitochondrial DNA.

Primer	Sequence 5' – 3'	References
LCO1490	GGTCAACAAATCATAAAGATATTGG	Folmer et al. 1994
HC02198	TAAACTTCAGGGTGACCAAAAAATCA	Folmer et al. 1994
16Sar-L	CGCCTGTTTATCAAAAACAT	Palumbi et al. 1991
16Sbr-H	CCGGTCTGAACTCAGATCACGT	Palumbi et al. 1991
144F	TGAGSNCARATGTCNTWYTG	Merritt et al. 1998
272R	GCRAANAGRAARTACCAYTC	Merritt et al. 1998
FCOII	AAATAATGCTATTTCATGAYCAYG	Hugall et al. 2002
RCOII	GCTCCGCAAATCTCTGARCAYTG	Hugall et al. 2002
1F_556 Os	TACCTGTACTAGCGGGGGCT	this paper
1R_75 Os	CAGTCAGGGTACTGCGGCTA	this paper
2F_342 Os	TTGTGACCTCGATGTTGGACT	this paper
2R_83 Os	CCGCCTCAGACCCAACTAAC	this paper
3F_320 Os	GGCCTAACTTGTTCACTGATCCT	this paper
3R_50 Os	TTTCTAGGGTCTGCGCTTCA	this paper
4F_429 Os	TTGTGGGGGTTTATTACGGGC	this paper
4R_110 Os	ATCACTCAACACCCCTGAAGT	this paper
seqF_F1	ACGGTTTCCTGTTCTATTATTTG	this paper
seqF_R1	CAAATAATAAGCTCCTAATGTAATC	this paper
seqF_R2	ATAAACTTTCCACTTCAGGGAAT	this paper
seqF_R3	GTAAAACATTTATTGGGGCCCAG	this paper
seqF_R4	AACTAATTAACAACCTATATAGGG	this paper
seqF_R5	TAGTCCCGTGCTGGCTAGTATT	this paper
seqH_F2	CTATTGTAACTCGCCTTAACTCTAA	this paper
seqH_R2	GAAATAAACACCTAAAATTACTGTA	this paper
seqH_R3	GATGTACCTGATATTAAACCTA	this paper
seqH_F4	CTACTAAACAGAAAAAGCGAACCC	this paper
seqH_R4	GCAGCCACAATTTACTTCTT	this paper

The mitogenome was annotated using the MITOS Web Server (Bernt et al. 2013). For the phylogenetic analysis we used a concatenated sequence alignment of 12 protein coding genes (PCGs; excluding *atp8*), and 2 rRNAs (*12S rRNA* and *16S rRNA*). Every set of 14 sequences was separately aligned using CLUSTAL W (Thompson et al. 1994) implemented in BIOEDIT v. 7.0.6 (Hall 1999; BioEdit 2017). The length of the alignment after combining the 14 gene sequences was for each species 14,287 bp. For the phylogenetic analysis we used all mitogenome sequences deposited in GenBank for species of the superfamily Helicoidea (Table 2). The mitogenome of *Thebapisana* (MH362760) was not annotated, so we designated the individual PCGs and rRNAs by aligning the whole *T.pisana* sequence with the extracted sequences of species belonging to the family Helicidae. Each of the *T.pisana*PCGs was tested for start and stop codons with ORF Finder (2004). Mitogenomes of two arionoid species (*Arionvulgaris* and *Meghimatiumbilineatum*, Table 2) were used as the outgroup.

**Table 2.** Mitogenomes from GenBank used in the phylogenetic analysis and their lengths.

species	GenBank Accession No.	Mitogenome length (bp)	References
Camaenidae: *Aegistaaubryana* (Heude, 1882)	KT192071	14238	Yang et al. 2016
Camaenidae: *Aegistadiversifamilia* Huang, Lee, Lin & Wu, 2014	KR002567	14039	Huang et al. 2016
Camaenidae: *Camaenacicatricosa* (O. F. Müller, 1774)	KM365408	13843	Wang et al. 2014
Camaenidae: *Camaenapoyuensis* Zhou, Wang & Ding, 2016	KT001074	13798	Lin et al. 2016
Camaenidae: *Dolicheulotaformosensis* (Adams, 1866)	KR338956	14237	Huang et al. 2016
Camaenidae: *Fruticicolakoreana* (L. Pfeiffer, 1850)	KU237291	13979	Hwang 2015
Camaenidae: *Mastigeulotakiangsinensis* (Martens, 1875)	KM083123	14029	Deng et al. 2016
Geomitridae: *Cernuellavirgata* (Da Costa, 1778)	KR736333	14147	Lin et al. 2016
Geomitridae: *Helicellaitala* (Linnaeus, 1758)	KT696546	13967	Romero et al. 2016
Helicidae: *Cylindrusobtusus* (Draparnaud, 1805)	JN107636	14610	Groenenberg et al. 2012
Helicidae: *Cepaeanemoralis* (Linnaeus, 1758)	U23045	14100	Terrett et al. 1996
Helicidae: *Cornuaspersum* (O. F. Müller, 1774)	JQ417194	14050	Gaitán-Espitia et al. 2013
Helicidae: *Helixpomatia* Linnaeus, 1758	MK347426	14070	Korabek et al. 2019
Helicidae: *Helixpomatia* Linnaeus, 1758	MK488030	14072	Groenenberg and Duijm 2019
Helicidae: *Helixpomatia* Linnaeus, 1758	MK488031	14070	Groenenberg and Duijm 2019
Helicidae: *Thebapisana* (O. F. Müller, 1774)	MH362760	14795	Wang et al. 2018
Hygromiidae: *Monachacartusiana* (O. F. Müller, 1774)	MW485067	13894	This paper
Polygyridae: *Practicolellamexicana* Perez, 2011 ^1^	KX278421	14008	Minton et al. 2016a
Polygyridae: *Practicolellamexicana* Perez, 2011 ^2^	KX240084	14153	Minton et al. 2016b
Arionidae: *Arionvulgaris* Moquin-Tandon, 1855	MN607980	14548	Doğan et al. 2020
Philomycidae: *Meghimatiumbilineatum* (Benson, 1842)	MG722906	14347	Yang et al. 2019

^1^ Deposited in GenBank as mitogenome of *Polygyracereolus* (Megerle von Mühlfeldt, 1818) but according to Minton et al. (2016a) it represents *Practicolellamexicana* Perez, 2011. ^2^ Mitogenome not mentioned in the paper by Minton et al. (2016a) but directly submitted to GenBank (Minton et al. 2016b).

**Table 3.** Organisation of the mitogenome of *Monachacartusiana.*

Type	Gene product	Gene acronym	Start	End	Length (bp)	Direction	Start codon	Stop codon
**PCG**	cytochrome c oxidase subunit I	*coI*	0	1552	1552	+	ATG	TAA^1^
**tRNA**	valine transfer RNA	*tRNA Val*	1525	1585	61	+		
**rRNA**	16S ribosomal RNA	*16S rRNA*	1242	2652	1410	+		
**tRNA**	leucine transfer RNA	*tRNA Leu*	2593	2657	65	+		
**tRNA**	proline transfer RNA	*tRNA Pro*	2654	2718	60	+		
**tRNA**	alanine transfer RNA	*tRNA Ala*	2716	2778	63	+		
**PCG**	NADH dehydrogenase subunit 6	*nd6*	2777	3263	451	+	ATT	TAA
**PCG**	NADH dehydrogenase subunit 5	*nd5*	3316	4915	1657	+	ATA	TAG
**PCG**	NADH dehydrogenase subunit 1	*nd1*	4896	5799	901	+	ATA	TAA^1^
**PCG**	NADH dehydrogenase subunit 4L	*nd4l*	5843	6076	233	+	TTG	TAT
**PCG**	cytochrome b	*cytb*	6054	7192	1097	+	GTC	TAA^1^
**tRNA**	aspartic acid transfer RNA	*tRNA Asp*	7192	7263	71	+		
**tRNA**	cysteine transfer RNA	*tRNA Cys*	7250	7310	61	+		
**tRNA**	phenylalanine transfer RNA	*tRNA Phe*	7310	7369	60	+		
**PCG**	cytochrome c oxidase subunit II	*coII*	7370	8052	672	+	ATG	TAA^1^
**tRNA**	tyrosine transfer RNA	*tRNA Tyr*	8040	8102	55	+		
**tRNA**	tryptophan transfer RNA	*tRNA Trp*	8094	8158	65	+		
**tRNA**	glycine transfer RNA	*tRNA Gly*	8158	8223	66	+		
**tRNA**	histidine transfer RNA	*tRNA His*	8216	8274	58	+		
**tRNA**	glutamine transfer RNA	*tRNA Gln*	8274	8331	57	-		
**tRNA**	leucine transfer RNA	*tRNA Leu*	8320	8392	73	-		
**PCG**	ATP synthase F0 subunit 8	*atp8*	8385	8544	104	-	ATG	TAA^1^
**tRNA**	asparagine transfer RNA	*tRNA Asn*	8544	8602	59	-		
**PCG**	ATP synthase F0 subunit 6	*atp6*	8582	9242	661	-	ATG	TAA
**tRNA**	arginine transfer RNA	*tRNA Arg*	9241	9304	62	-		
**tRNA**	glutamic acid transfer RNA	*tRNA Glu*	9303	9367	65	-		
**rRNA**	12S ribosomal RNA	*12S rRNA*	9412	10120	798	-		
**tRNA**	metionine transfer RNA	*tRNA Met*	10118	10180	63	-		
**PCG**	NADH dehydrogenase subunit 3	*nd3*	10160	10493	307	-	ATT	TAA^1^
**tRNA**	serine transfer RNA	*tRNA Ser*	10523	10576	53	-		
**tRNA**	serine transfer RNA	*tRNA Ser*	10648	10700	52	+		
**PCG**	NADH dehydrogenase subunit 4	*nd4*	10721	12005	1210	+	ATT	TAG
**tRNA**	threonine transfer RNA	*tRNA Thr*	11996	12058	63	-		
**PCG**	cytochrome c oxidase subunit III	*coIII*	12046	12833	776	-	ATG	TAA^1^
**tRNA**	isoleucine transfer RNA	*tRNA Ile*	12877	12937	61	+		
**PCG**	NADH dehydrogenase subunit 2	*nd2*	12899	13872	833	+	ATA	TAA^1^
**tRNA**	lysine transfer RNA	*tRNA Lys*	13842	13894	60	+		

^1^ Stop codons completed by the addition of 3' A residues to mRNA.

Phylogenetic analysis was performed using maximum likelihood (ML) as implemented in the online version of IQ-TREE (Trifinopoulos et al. 2016). ML analysis was done using 14 partitions. Best substitution models were inferred according to the Bayesian information criterion (BIC) for each of the partitions by ModelFinder (Kalyaanamoorthy et al. 2017) implemented in IQ-TREE. The TVM+F+I+G4 model was selected for *nd1*, *nd2*, *nd4*, *nd5*, *atp6*, and *16S rRNA*; TPM3u+F+I+G4 for *nd3*; K3Pu+F+G4 for *nd4l*; TPM3+F+I+G4 for *nd6*; K3Pu+F+I+G4 for *cytb*, and *coII*; TIM+F+I+G4 for *coI*; GTR+F+I+G4 for *coIII*, and *12S rRNA*. ML trees were constructed under 1,000 ultrafast bootstrap replicates (Minh et al. 2013) and with Shimodaira-Hasegawa-like approximate likelihood ratio test with 1,000 replicates (SH-aLRT; Guindon et al. 2010). A Bayesian inference (BI) analysis was performed with MrBayes v. 3.2.6 (Ronquist et al. 2012). Four Monte Carlo Markov chains were run for 1 million generations, sampling every 100 generations (the first 25% of trees were discarded as “burn-in”). Ultrafast bootstrap support, SH-aLRT support (both expressed in percentages) and posterior probability (PP) values obtained on 50% majority rule consensus Bayesian tree were mapped on the ML tree of concatenated sequences. The ML tree was visualized using FigTree v. 1.4.3 (Rambaut 2016).

## ﻿Results and discussion

**Figure 1.**
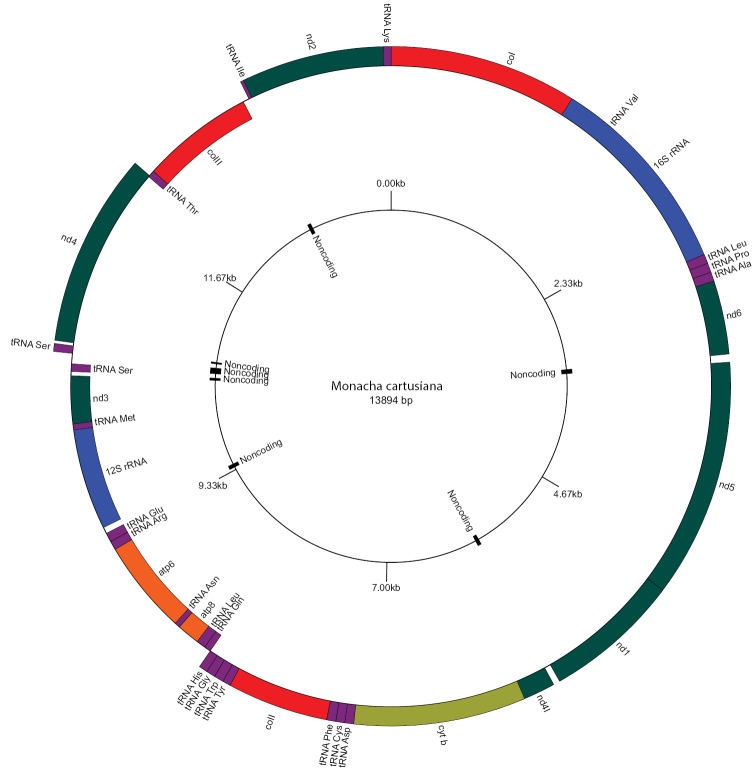
Circular diagram of the mitochondrial genome of *Monachacartusiana* (GenBank acc. no. MW485067). Genes encoded in the “plus” and the “minus” directions are shown outside and inside the circle, respectively. Particular gene types are marked with different colours: red – PCGs coding I, II, and III subunits of cytochrome c oxidase; green – PCGs coding NADH dehydrogenase family; orange – PCGs coding ATPase family; yellow – sequence coding cytochrome b; purple – tRNAs coding sequences; blue – rRNA coding genes. Noncoding sequences are mapped on a small inner circle. The circular diagram was created with GenomeVx (Conant and Wolfe 2008).

The complete mitogenome of *M.cartusiana* was deposited in GenBank under accession number MW485067. With 13,894 bp in length, it was one of the shortest mito­genomes known in Helicoidea, which ranged from 13,798 bp (*Camaenapoyuensis*) to 14,795 bp (*Thebapisana*) (Table 2). The mitogenome included: 13 PCGs, 22 tRNA genes and two rRNA genes (Fig. 1, Table 3), typical for most metazoan mitogenomes. The base composition of the *M.cartusiana* mitogenome was: 30.26% A, 37.95% T, 16.94% G and 14.85% C, i.e. with a bias towards A and T (68.21% content of A-T). These values differ from other helicoid species, but fit into the range previously reported for helicoids, especially when compared with the A-T values for *C.virgata* (65.96%) and *H.itala* (66.22%) (Doğan et al. 2020: table S3). The total length of all PCGs was 10,404 bp (74.88% of the entire mitogenome), and they had different start and stop codons, which also vary among helicoid mitogenomes (Table 4). Some of the stop codons TAA were generated by posttranscriptional polyadenylation (as in Groenenberg et al. 2012 and Yang et al. 2016). Nine PCGs were encoded in the “plus” direction (*nd1*, *nd2*, *nd4*, *nd4l*, *nd5*, *nd6*, *cytb*, *coI*, *coII*) and four in the “minus” direction (*coIII*, *atp6*, *atp8*, *nd3*). Furthermore, 14 tRNA and one rRNA were encoded in the “plus” direction and eight tRNA and one rRNA in the “minus” direction (Table 3). Additionally, seven intergenic regions (with noncoding sequences) were identified with a total length of 295 bp (the longest was 70 bp while the shortest 19 bp) (Fig. 1). The gene order in *M.cartusiana* mitogenome was exactly the same as in *C.virgata* and *H.itala* (geomitrid species). Yet, the polygyrid *Practicolellamexicana* differed in four places and helicid species in seven (Table 5). The species representing the Camaenidae formed three groups with the same order of genes, but each of these groups differed in gene order from species from Hygromiidae, Geomitridae, Helicidae, and Polygyridae (Table 5).

**Table 4.** Start and stop codons in the mitogenome protein coding genes of helicoid species.

Species	Start codons	Stop codons
*Monachacartusiana*	ATA – 3; ATG – 5; ATT – 3; GTC – 1; TTG – 1	TAA – 10; TAG – 2; TAT – 1
*Cernuellavirgata*	ATA – 4; ATG – 4; ATT – 5	TAA – 9; TAG – 4
*Helixpomatia*	ATA – 1; ATC – 1; ATG – 6; GTG – 3; TTG – 2	TAA – 8; TAG – 5
*Cepaeanemoralis*	ATA – 5; ATG – 2; ATT – 6	TAA – 2; TAG – 4; TA – 7
*Cornuaspersum*	ATA – 5; ATG – 6; TTG – 2	TAA – 5; TAG – 5; T – 3
*Thebapisana*	ATA – 2; ATC – 1; ATG – 2; ATT – 8	TAA/TAG – 12; T – 1
*Cylindrusobtusus*	ATA – 5; ATG – 4; ATC – 1; GTG – 1; TTG – 2	TAA – 4; TAG – 5; T – 4
*Practicolellamexicana*	ATC – 1; ATG – 5; ATT – 2; GTG – 2; TTG – 3	TAA – 3, TAG – 4; T – 6
*Aegistaaubryana*	ATA – 6; ATG – 7	TAA/TAG – 11; T – 2
*Aegistadiversifamilia*	ATG – 5; ATT – 3; TTG – 3; TTA – 2	TAA – 5; TAG – 2; TA – 2; T – 4
*Camaenacicatricosa*	ATA – 5; ATG – 4; ATT – 3; GTG – 1	TAA – 11; TAG – 2
*Dolicheulotaformosensis*	ATG – 4; ATA – 3; ATT – 3; TTG – 2; GTG – 1	TAA – 5; TAG – 2; TA – 6
*Mastigeulotakiangsinensis*	ATA – 4; ATG – 7; ATT – 1; GTG – 1	TAA – 7; TAG – 6

For references see Table 2.

**Table 5.** Gene order in known mitogenomes of helicoid species.

	*M.cartusiana*	*coI*	*Val*	*16S*	*Leu*	*Pro*	*Ala*	*nd6*	*nd5*	*nd1*	*nd4l*	*cytb*	*Asp*	*Cys*	*Phe*	*coII*	*Tyr*	*Trp*	*Gly*	*His*	*Gln*	*Leu*	*atp8*	*Asn*	*atp6*	*Arg*	*Glu*	*12S*	*Met*	*nd3*	*Ser*	*Ser*	*nd4*	*Thr*	*coIII*	*Ile*	*nd2*	*Lys*
	*C.virgata*	*coI*	*Val*	*16S*	*Leu*	*Pro*	*Ala*	*nd6*	*nd5*	*nd1*	*nd4l*	*cytb*	*Asp*	*Cys*	*Phe*	*coII*	*Tyr*	*Trp*	*Gly*	*His*	*Gln*	*Leu*	*atp8*	*Asn*	*atp6*	*Arg*	*Glu*	*12S*	*Met*	*nd3*	*Ser*	*Ser*	*nd4*	*Thr*	*coIII*	*Ile*	*nd2*	*Lys*
	*H.itala*	*coI*	*Val*	*16S*	*Leu*	*Pro*	*Ala*	*nd6*	*nd5*	*nd1*	*nd4l*	*cytb*	*Asp*	*Cys*	*Phe*	*coII*	*Tyr*	*Trp*	*Gly*	*His*	*Gln*	*Leu*	*atp8*	*Asn*	*atp6*	*Arg*	*Glu*	*12S*	*Met*	*nd3*	*Ser*	*Ser*	*nd4*	*Thr*	*coIII*	*Ile*	*nd2*	*Lys*
	*P.mexicana*	*coI*	*Val*	*16S*	*Leu*	*Pro*	*Ala*	*nd6*	*nd5*	*nd1*	*nd4l*	*cytb*	*Asp*	*Cys*	*Phe*	*coII*	*Gly*	*His*	*Tyr*	*Trp*	*Gln*	*Leu*	*atp8*	*Asn*	*atp6*	*Arg*	*Glu*	*12S*	*Met*	*nd3*	*Ser*	*Ser*	*nd4*	*Thr*	*coIII*	*Ile*	*nd2*	*Lys*
	*H.pomatia*	*coI*	*Val*	*16S*	*Leu*	*Ala*	*nd6*	*Pro*	*nd5*	*nd1*	*nd4l*	*cytb*	*Asp*	*Cys*	*Phe*	*coII*	*Tyr*	*Trp*	*Gly*	*His*	*Gln*	*Leu*	*atp8*	*Asn*	*atp6*	*Arg*	*Glu*	*12S*	*Met*	*nd3*	*Ser*	*Thr*	*coIII*	*Ser*	*nd4*	*Ile*	*nd2*	*Lys*
	*C.aspersum*	*coI*	*Val*	*16S*	*Leu*	*Ala*	*nd6*	*Pro*	*nd5*	*nd1*	*nd4l*	*cytb*	*Asp*	*Cys*	*Phe*	*coII*	*Tyr*	*Trp*	*Gly*	*His*	*Gln*	*Leu*	*atp8*	*Asn*	*atp6*	*Arg*	*Glu*	*12S*	*Met*	*nd3*	*Ser*	*Thr*	*coIII*	*Ser*	*nd4*	*Ile*	*nd2*	*Lys*
	*C.nemoralis*	*coI*	*Val*	*16S*	*Leu*	*Ala*	*nd6*	*Pro*	*nd5*	*nd1*	*nd4l*	*cytb*	*Asp*	*Cys*	*Phe*	*coII*	*Tyr*	*Trp*	*Gly*	*His*	*Gln*	*Leu*	*atp8*	*Asn*	*atp6*	*Arg*	*Glu*	*12S*	*Met*	*nd3*	*Ser*	*Thr*	*coIII*	*Ser*	*nd4*	*Ile*	*nd2*	*Lys*
	*C.obtusus*	*coI*	*Val*	*16S*	*Leu*	*Ala*	*nd6*	*Pro*	*nd5*	*nd1*	*nd4l*	*cytb*	*Asp*	*Cys*	*Phe*	*coII*	*Tyr*	*Trp*	*Gly*	*His*	*Gln*	*Leu*	*atp8*	*Asn*	*atp6*	*Arg*	*Glu*	*12S*	*Met*	*nd3*	*Ser*	*Thr*	*coIII*	*Ser*	*nd4*	*Ile*	*nd2*	*Lys*
	*Ae.aubryana*	*coI*	*Val*	*16S*	*Leu*	*Pro*	*Ala*	*nd6*	*nd5*	*nd1*	*nd4l*	*cytb*	*Asp*	*Cys*	*Phe*	*coII*	*Gly*	*His*	*Tyr*	*nd3*	*Trp*	*Gln*	*Leu*	*atp8*	*Asn*	*atp6*	*Arg*	*Glu*	*12S*	*Met*	*Ser*	*Ser*	*nd4*	*Thr*	*coIII*	*Ile*	*nd2*	*Lys*
	*Ae.diversifamilia*	*coI*	*Val*	*16S*	*Leu*	*Pro*	*Ala*	*nd6*	*nd5*	*nd1*	*nd4l*	*cytb*	*Asp*	*Cys*	*Phe*	*coII*	*Gly*	*His*	*Tyr*	*nd3*	*Trp*	*Gln*	*Leu*	*atp8*	*Asn*	*atp6*	*Arg*	*Glu*	*12S*	*Met*	*Ser*	*Ser*	*nd4*	*Thr*	*coIII*	*Ile*	*nd2*	*Lys*
	*D.formosensis*	*coI*	*Val*	*16S*	*Leu*	*Pro*	*Ala*	*nd6*	*nd5*	*nd1*	*nd4l*	*cytb*	*Asp*	*Cys*	*Phe*	*coII*	*Gly*	*His*	*Tyr*	*Trp*	*Gln*	*Leu*	*atp8*	*Asn*	*atp6*	*Arg*	*Glu*	*12S*	*Met*	*nd3*	*Ser*	*Ser*	*nd4*	*Thr*	*coIII*	*Ile*	*nd2*	*Lys*
	*F.koreana*	*coI*	*Val*	*16S*	*Leu*	*Pro*	*Ala*	*nd6*	*nd5*	*nd1*	*nd4l*	*cytb*	*Asp*	*Cys*	*Phe*	*coII*	*Gly*	*His*	*Tyr*	*Trp*	*Gln*	*Leu*	*atp8*	*Asn*	*atp6*	*Arg*	*Glu*	*12S*	*Met*	*nd3*	*Ser*	*Ser*	*nd4*	*Thr*	*coIII*	*Ile*	*nd2*	*Lys*
	*M.kiangsinensis*	*coI*	*Val*	*16S*	*Leu*	*Pro*	*Ala*	*nd6*	*nd5*	*nd1*	*nd4l*	*cytb*	*Asp*	*Cys*	*Phe*	*coII*	*Gly*	*His*	*Tyr*	*Trp*	*Gln*	*Leu*	*atp8*	*Asn*	*atp6*	*Arg*	*Glu*	*12S*	*Met*	*nd3*	*Ser*	*Ser*	*nd4*	*Thr*	*coIII*	*Ile*	*nd2*	*Lys*
	*C.cicatricosa*	*coI*	*Val*	*16S*	*Leu*	*Pro*	*Ala*	*nd6*	*nd5*	*nd1*	*nd4l*	*cytb*	*Cys*	*Phe*	*coII*	*Asp*	*Tyr*	*Gly*	*His*	*Trp*	*Gln*	*Leu*	*atp8*	*Asn*	*atp6*	*Arg*	*Glu*	*12S*	*Met*	*nd3*	*Ser*	*Ser*	*nd4*	*Thr*	*coIII*	*Ile*	*nd2*	*Lys*
	*C.poyuensis*	*coI*	*Val*	*16S*	*Leu*	*Pro*	*Ala*	*nd6*	*nd5*	*nd1*	*nd4l*	*cytb*	*Cys*	*Phe*	*coII*	*Asp*	*Tyr*	*Gly*	*His*	*Trp*	*Gln*	*Leu*	*atp8*	*Asn*	*atp6*	*Arg*	*Glu*	*12S*	*Met*	*nd3*	*Ser*	*Ser*	*nd4*	*Thr*	*coIII*	*Ile*	*nd2*	*Lys*

**Figure 2.**
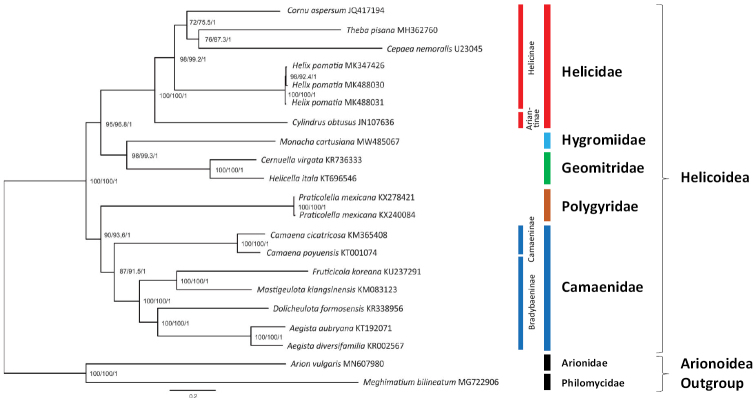
Maximum likelihood (ML) tree of mitochondrial genomes of species representing the superfamily Helicoidea (see Table 1). Mitogenome sequences included all PCGs (except *atp8*) and two rRNA genes were 14,287 positions in length. Ultrafast bootstrap support values (%), SH-aLRT support values (%) and Bayesian posterior probabilities are indicated next to the branches. The tree was rooted with sequences of *Arionvulgaris* (MN607980) and *Meghimatiumbilineatum* (MG722906) mitogenomes deposited in GenBank by Doğan et al. (2020) and Yang et al. (2019), respectively.

Light blue background shows the same position in gene order as in *M.cartusiana* mitogenome. For gene acronyms (tRNA genes shortened to aminoacid symbol) and references see Table 2. Colours for the families as in Fig. 2: light blue – Hygromiidae; green – Geomitridae; brown – Polygyridae; red – Helicidae; dark blue – Camaenidae

Phylogenetic analyses of the stylommatophoran mitogenomes (González et al. 2016; Romero et al. 2016) showed them in a well-supported clade among Panpulmonata (with PP and bootstrap values 1 and 99, respectively). Previous mitogenome phylogenies of stylommatophoran superfamilies (Groenenberg et al. 2017; Harasewych et al. 2017; Yang et al. 2019; Doğan et al. 2020) showed a clade of Helicoidea separate from other superfamilies, although mitogenomes of only 11 stylommatophoran superfamilies (Yang et al. 2019) out of 26 listed by Bouchet et al. (2017) are represented in GenBank. According to Bouchet et al. (2017), Helicoidea includes 17 families but hitherto phylogenetic relationships could be analysed only for three or four of them, namely Helicidae, Camaenidae, Geomitridae, and Polygyridae (González et al. 2016; Lin et al. 2016; Minton et al. 2016a; Harasewych et al. 2017; Doğan et al. 2020).

For the phylogenetic analysis, a concatenated alignment of 12 PCGs (excluding *atp8*, because it was too short, too variable, and not annotated in the mitogenome of *Cernuellavirgata*) and 2 rRNAs (12S and 16S) was used. The dataset included 19 helicoid species (Table 2) yielding the ML tree shown in Fig. 2. The Bayesian tree (not shown) had the same topology.

The mitogenome of *M.cartusiana* allows to add Hygromiidae to the previous analyses of Helicoidea families. It shows up in a clade with mitogenomes of the geomitrid species, *Cernuellavirgata* and *Helicellaitala*, confirming the close relationships of two families, i.e., Hygromiidae and Geomitridae (Razkin et al. 2015). The mitogenome of the helicid *Cylindrusobtusus* of the subfamily Ariantinae forms a branch separated from the subfamily Helicinae (Fig. 2). This was also noted in previous phylogenetic analyses (Korábek et al. 2019; Doğan et al. 2020). Moreover, Camaenidae are separated into two clades i.e., Bradybaeninae and Camaeninae, treated frequently as two separate families (Lin et al. 2016; Minton et al. 2016a; Harasewych et al. 2017). Our results agree with the division of Helicidae and Camaenidae into subfamilies (Bouchet at al. 2017). However, the five helicid and seven camaenid species (Table 2, Fig. 2) represent only a tiny fraction of these speciose families. Therefore, more helicoid and stylommatophoran mitogenomes are urgently needed.
